# Clinical Impact of New-Onset Left Bundle Branch Block After Transcatheter Aortic Valve Replacement: Data from a Single-Center Retrospective Registry

**DOI:** 10.21470/1678-9741-2024-0187

**Published:** 2025-05-23

**Authors:** Aleksey A. Baranov, Aram G. Badoian, Dmitrii A. Khelimskii, Aryuna Yu. Tsydenova, Ivan S. Peregudov, Vladimir V. Beloborodov, Aleksey G. Filippenko, Toyche U. Khalkhozhaev, Oleg V. Krestyaninov

**Affiliations:** 1 Meshalkin National Medical Research Center, Ministry of Health of Russian Federation, Novosibirsk, Russian Federation

**Keywords:** Aortic Stenosis, Transcatheter Aortic Valve Replacement, Left Bundle Branch Block, Permanent Pacemaker Implantation

## Abstract

**Introduction:**

The clinical significance of new-onset left bundle branch block (LBBB) after transcatheter aortic valve replacement (TAVR) remains controversial. In the presented study, we aimed to assess the impact of new LBBB on clinical outcomes after TAVR.

**Methods:**

A total of 473 patients underwent TAVR for severe aortic stenosis between 2015 and 2023. According to the exclusion criteria, the study cohort comprised of 322 patients for analysis. The primary endpoint was cardiovascular death, with secondary endpoints including all-cause mortality and permanent pacemaker implantation (PPI) during follow-up.

**Results:**

Patients with new LBBB had a significantly smaller indexed aortic valve area (0.3 ± 0.1 vs. 0.4 ± 0.1, P < 0.01) and interventricular membranous septum length (6.2 ± 1.6 vs. 6.9 ± 1.8, P < 0.01). By multivariable analysis, new LBBB remained an independent predictor of cardiovascular death (hazard ratio [HR] 7.09, 95% confidence interval [CI] 1.16 - 43.50, P = 0.03) during the 2.9-year follow-up period. There were no significant differences in the incidence of all-cause mortality (HR 0.48, 95% CI 0.17 - 1.37, P = 0.16) and PPI (HR 2.61, 95% CI 0.85 - 0.80, P = 0.08) between patients with new LBBB compared to those without it.

**Conclusion:**

New LBBB after TAVR procedure is associated with an increased risk of death from cardiovascular causes, but it did not increase the risk of all-cause mortality and PPI over the long-term period.

## INTRODUCTION

**Table:** 

Abbreviations, Acronyms & Symbols				
AR	= Aortic regurgitation		LBBB	= Left bundle branch block
AS	= Aortic stenosis		LV	= Left ventricular
AV	= Aortic valve		MI	= Myocardial infarction
AVB	= Atrioventricular block		MR	= Mitral regurgitation
BAV	= Balloon aortic valvuloplasty		MS	= Membranous septum
BMI	= Body mass index		NYHA	= New York Heart Association
CABG	= Coronary artery bypass grafting		OR	= Odds ratio
CI	= Confidence interval		OT	= Outflow tract
COPD	= Chronic obstructive pulmonary disease		PCI	= Percutaneous coronary intervention
ECG	= Electrocardiogram		PPI	= Permanent pacemaker implantation
EF	= Ejection fraction		RBBB	= Right bundle branch block
EuroSCORE	= European System for Cardiac Operative Risk Evaluation		SD	= Standard deviation
GFR	= Glomerular filtration rate		STS	= Society of Thoracic Surgeons
HR	= Hazard ratio		STS-PROM	= Society of Thoracic Surgeons Predicted Risk of Mortality
IMMLV	= Indexed myocardial mass of left ventricle		TAVR	= Transcatheter aortic valve replacement
IQR	= Interquartile range		TR	= Tricuspid regurgitation

Transcatheter aortic valve replacement (TAVR) is an effective alternative to surgical aortic valve replacement for symptomatic severe aortic stenosis (AS) in patients at high, moderate, and even low surgical risk^[1^-^3]^. Advances in surgical proficiency and the enhancement of transcatheter valves have notably minimized the occurrence of procedural complications. Nonetheless, a significant challenge remains due to the high incidence of postoperative atrioventricular conduction disorders. The emergence of periprocedural cardiac conduction disorders during or immediately after TAVR is primarily attributed to direct mechanical damage to the components of the cardiac conduction system located in close proximity to the aortic valve^[4]^. New-onset left bundle branch block (LBBB) is the most common type of cardiac conduction disorder after TAVR, with incidence rates ranging from 4% to 30% for balloon-expandable and 18% to 65% for self-expanding valves^[5^,^6]^. While there is extensive research on the occurrence and predictors of new conduction disturbances following TAVR, the available data on the potential prognostic significance of new-onset LBBB are limited and controversial^[7^,^8]^. Few studies have shown competing results regarding the association between new-onset post-TAVR LBBB and a higher increased all-cause and cardiovascular mortality, rehospitalization, as well as a greater need for permanent pacemaker implantation (PPI)^[9^,^10]^. However, several other studies have failed to demonstrate an association between new LBBB and mortality^[11^,^12]^. There is currently a scarcity of data regarding the impact of new-onset LBBB on myocardial remodeling and cardiac contractile function after TAVR. Considering the expanding use of TAVR for younger patients, the current issue is even more important. This study aimed to assess the effect of new-onset LBBB on long-term outcomes following TAVR.

## METHODS

This retrospective study initially included 441 patients who underwent TAVR for severe AS at a single center from March 2015 to October 2023. Patients with preexisting cardiac conduction abnormalities (QRS > 120 ms, LBBB, right bundle branch block) (52 patients) and those who received a permanent pacemaker either before or immediately after the index procedure during the same hospitalization (59 patients) were excluded from the analysis. Additionally, eight cases resulting in hospital mortality were also excluded. Therefore, the final analysis included 322 patients. The indications for TAVR were determined by current European Society of Cardiology/European Association for CardioThoracic Surgery guidelines for the treatment of valvular heart disease: 1) mean aortic gradient ≥ 40 mmHg; or 2) peak velocity ≥ 4.0 m/s^[13]^. Surgical risk was assessed using the European System for Cardiac Operative Risk Evaluation (or EuroSCORE) II and the Society of Thoracic Surgeons Predicted Risk of Mortality (or STS-PROM)^[14^,^15]^. LBBB was diagnosed based on electrocardiogram (ECG) findings showing wide QRS complexes >120 ms in the left-sided leads V5 and V6, as well as a notched R wave in leads I, aVL, V5, and V6^[16]^. In the present study, new-onset LBBB was considered persistent if it emerged during or following the TAVR procedure and was recorded on the ECG upon hospital discharge or within seven days post-TAVR. The primary endpoint was a cardiovascular death. The secondary endpoint included all-cause mortality as well as PPI. All clinical outcomes were analyzed according to the Valve Academic Research Consortium (or VARC-3)^[17]^.

### Statistical Analysis

All statistical tests were two-tailed, and *P*-values < 0.05 were considered statistically significant. Statistical analyses were performed using R Statistical Software (version 4.3.1; R Foundation for Statistical Computing, Vienna, Austria) and RStudio (version 2023.06.1 Build 524). Continuous variables are presented as mean ± standard deviation when normally distributed and as median and interquartile range - 25^th^ and 75^th^ percentiles for variables with non-normal distribution. Categorical variables were presented as absolute number and percentage. For the between-group comparison of continuous variables, the Student's *t*-test was used. Non-parametric Mann-Whitney U test was used when comparing non-normally distributed continuous variables. Between-group comparison of categorical variables was performed using Fisher's exact test. To determine predictors of death from cardiovascular causes, multivariate Cox regression analysis was performed, which included all parameters that could potentially be associated with the primary endpoint as independent variables. Time-to-event analysis was performed using the Kaplan-Meier method, and between-group differences were checked by log-rank test. Differences were considered statistically significant at *P* ≤ 0.05. The study was approved by the Meshalkin National Medical Research Center ethics committee (n. 06-5) and complies with the principles of the Declaration of Helsinki.

## RESULTS

In the overall patient population, the incidence of new LBBB after TAVR was 20.2%. Baseline clinical and instrumental characteristics of patients are presented in Table 1. In patients with new LBBB, there was a significantly smaller indexed aortic valve area (0.3 ± 0.1 mm *vs.* 0.4 ± 0.1 mm, *P* < 0.01) and interventricular membranous septum length (6.2 ± 1.6 mm *vs.* 6.9 ± 1.8 mm, *P* < 0.01).

**Table 1:** Baseline characteristics.

Parameters	New LBBB n=65	No LBBB n=257	*P*-value
	Mean (± SD)/N (%)/Median (IQR)		
Male sex	29 (44.6)	101 (39.3)	0.44
Age	74.2 ± 8.6	75.7 ± 7.0	0.14
BMI	29.2 ± 5.4	30.6 ± 6.4	0.11
NYHA III-IV	59 (90.8)	207 (80.5)	0.05
Atrial fibrillation or flutter	13 (20.0)	69 (26.8)	0.26
MI	14 (21.5)	64 (24.9)	0.57
Oncopathology	6 (9.2)	37 (14.4)	0.27
Stroke	7 (10.8)	20 (7.8)	0.44
Diabetes mellitus	16 (24.6)	77 (30.0)	0.40
COPD	7 (10.8)	31 (12.1)	0.77
Prior PCI	22 (33.8)	107 (41.6)	0.25
Prior CABG	7 (10.8)	24 (9.3)	0.73
Prior BAV	4 (6.2)	19 (7.4)	0.73
EuroSCORE II	5.8 ± 4.8	6.3 ± 5.2	0.40
STS score	3.1 ± 2.0	3.2 ± 2.0	0.72
GFR	70.4 ± 16.1	68.3 ± 16.1	0.35
AV mean gradient	52.9 ± 15.1	55.8 ± 16.4	0.20
Indexed AV area	0.3 ± 0.1	0.4 ± 0.1	< 0.01
LV EF	59.1 ± 12.0	58.9 ± 12.3	0.91
Bicuspid AV	3 (4.6)	24 (9.3)	0.22
IMMLV	168.9 ± 41.9	170.1 ± 47.2	0.85
Prior mitral regurgitation (moderate/severe)	15 (23.1)	79 (30.7)	0.22
Prior aortic regurgitation (moderate/severe)	6 (9.2)	37 (14.4)	0.27
Prior tricuspid regurgitation (moderate/severe)	2 (3.1)	15 (5.8)	0.37
Prior QRS	95.2 ± 12.9	98.4 ± 23.2	0.29
First-degree AVB	3 (4.2)	24 (9.3)	0.22
Incomplete RBBB	5 (7.7)	20 (7.8)	0.98
Left anterior hemiblock	5 (7.7)	32 (12.5)	0.28
Left posterior hemiblock	0 (0)	2 (0.8)	0.48
Aortic root angle	48.2 ± 9.3	48.8 ± 8.0	0.60
Calcification of LV OT	2 (3.1)	23 (8.9)	0.11
Membranous septum length	6.0 (5.0;7.1)	6.9 (5.7;8.0)	< 0.01

AV=aortic valve; AVB=atrioventricular block; BAV=balloon aortic valvuloplasty; BMI=body mass index; CABG=coronary artery bypass grafting; COPD=chronic obstructive pulmonary disease; EF=ejection fraction; EuroSCORE=European System for Cardiac Operative Risk Evaluation; GFR=glomerular filtration rate; IMMLV=indexed myocardial mass of left ventricle; IQR=interquartile range; LBBB=left bundle branch block; LV=left ventricular; MI=myocardial infarction; NYHA=New York Heart Association; OT=outflow tract; PCI=percutaneous coronary intervention; RBBB=right bundle branch block; SD=standard deviation; STS=Society of Thoracic Surgeons

Procedural results are presented in Table 2. The use of the first-generation CoreValve® prosthesis was more frequent among patients with new LBBB compared with patients without LBBB (53.8% *vs.* 36.6%, *P* = 0.01). Moreover, the groups were significantly different in implantation depth (6.3 ± 2.4 mm in the new LBBB group and 4.6 ± 2.4 mm in the no LBBB group, *P* < 0.01) and in the arithmetic difference between membranous septum length and implantation depth (0.2 [-1.6;1.3] in the new LBBB group *vs.* 1.8 [0.25;4.1] in the no LBBB group, *P*<0.01). There were no significant differences in preor post-dilatation between the two groups. Similarly, the frequency of procedural failure and duration of post-TAVR hospitalization did not differ significantly.

**Table 2:** Procedural results.

Parameters		New LBBB n=65	No LBBB n=257	*P*-value
		Mean (± SD)/N (%)		
Pre-dilatation		59 (90.8)	236 (91.8)	0.08
Post-dilatation		30 (46.2)	134 (52.1)	0.39
Bioprosthesis type	CoreValve®	35 (53.8)	94 (36.6)	0.01
	Evolute™ R	22 (33.9)	80 (31.1)	0.67
	ACURATE neo™	6 (9.2)	76 (29.6)	< 0.01
	ACURATE neo2™	2 (3.1)	7 (2.7)	0.88
Prosthesis size	23	8 (12.3)	16 (6.2)	0.09
	25	5 (7.7)	43 (16.7)	0.06
	26	10 (15.4)	44 (17.1)	0.74
	27	4 (6.2)	34 (13.2)	0.11
	29	22 (33.8)	77 (30.0)	0.54
	31	7 (10.8)	17 (6.6)	0.26
	34	9 (13.8)	26 (10.2)	0.38
Implantation depth		6.3 ± 2.4	4.6 ± 2.4	< 0.01
MS length, implantation depth		0.2 (-1.6;1.3)	1.8 (0.25;4.1)	< 0.01
MR 2-3 after procedure		3 (4.6)	22 (8.6)	0.44
Postoperative QRS		141.3 ± 21.5	102.3 ± 19.9	< 0.01
Postoperative AV mean gradient		8.5 ± 4.0	8.8 ± 4.3	0.61
Procedural unsuccess		1 (1.5)	12 (4.7)	0.48
Time to discharge post-TAVR		9.4 ± 3.5	8.7 ± 4.2	0.22

AV=aortic valve; LBBB=left bundle branch block; MR=mitral regurgitation; MS=membranous septum; SD=standard deviation; TAVR= transcatheter aortic valve replacement

**Fig. 1:**
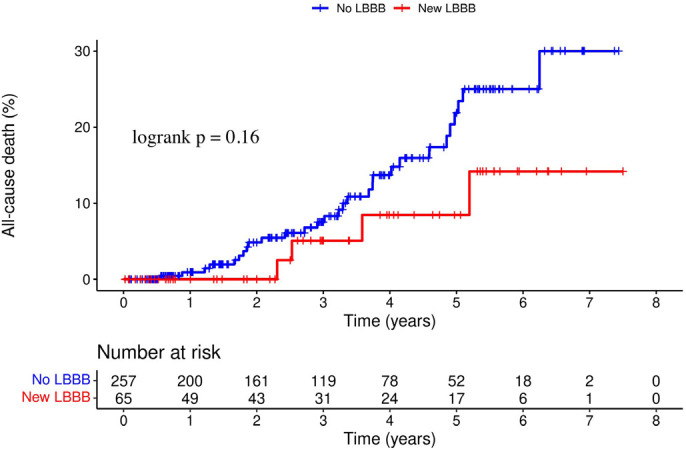
Kaplan-Meier curves of the incidence of all-cause death. LBBB=left bundle branch block.

**Fig. 2:**
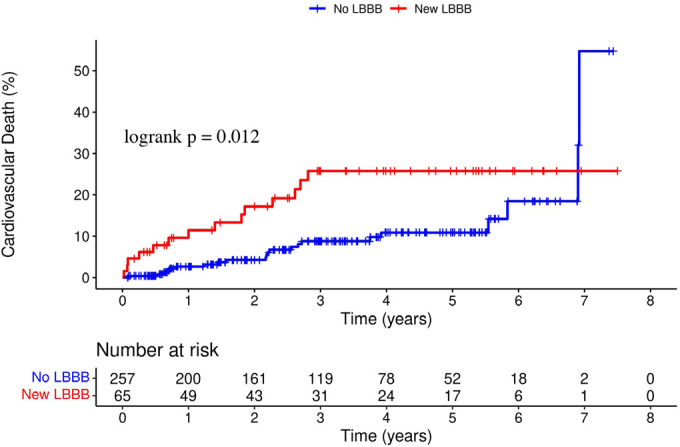
Kaplan-Meier curves of the incidence of death from cardiovascular causes. LBBB=left bundle branch block.

**Fig. 3:**
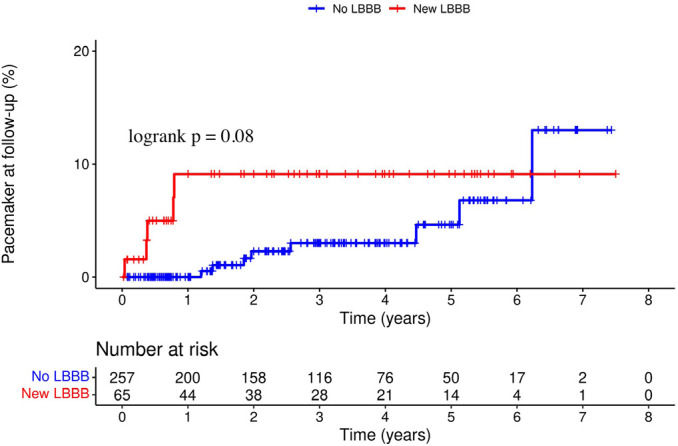
Kaplan-Meier curves of the incidence of permanent pacemaker implantation. LBBB=left bundle branch block.

At a mean follow-up of 2.9 ± 1.9 years, a total of 69 patients (21.4%) had died; there were 36 cases (11.2%) of cardiovascular death. A total of 18 patients (27.7%) with new LBBB died during the study period, 14 (21.5%) from cardiovascular causes. There were no significant differences between new LBBB and no LBBB groups in the incidence of death from all causes (27.7% *vs.* 19.8%, hazard ratio [HR] 0.48, 95% confidence interval [CI] 0.17 - 1.37, *P*=0.16) (Figure 1). In contrast, patients with new LBBB after TAVR had significantly higher rates of cardiovascular mortality (21.5% *vs.* 8.6%, HR 2.31, 95% CI 1.18 - 4.53, *P*=0.012) (Figure 2). In addition, there were no significant differences between new LBBB and no LBBB groups in the incidence of PPI during the follow-up (7.7% *vs.* 3.1%, HR 2.61, 95% CI 0.85 - 0.80, *P*=0.08) (Figure 3). All post-TAVR outcomes and HRs for adverse clinical events are described in Table 3. Multivariate regression analysis revealed age (odds ratio [OR] 1.26, 95% CI 1.05 - 1.51, *P* = 0.01), baseline left ventricular (LV) ejection fraction (EF) (OR 0.91, 95% CI 0.84 - 0.98, *P* = 0.02), and new-onset post-TAVR LBBB (OR 7.09, 95% CI 1.16 - 43.50, *P* = 0.03) as independent predictors of death from cardiovascular causes (Table 4).

**Table 3:** Follow-up outcomes.

Event	New LBBB n=65	No LBBB n=257	HR (95% CI)	*P*-value
	N (%)			
Death from cardiovascular causes	14 (21.5)	22 (8.6)	2.31 (1.18 - 4.53)	0.012
Death from all causes	18 (27.7)	51 (19.8)	0.48 (0.17 - 1.37)	0.16
Permanent pacemaker implantation	5 (7.7)	8 (3.1)	2.61 (0.85 - 0.80)	0.08

CI=confidence interval; HR=hazard ratio; LBBB=left bundle branch block

**Table 4:** Multivariate regression analysis of predictors of death from cardiovascular causes.

Factor	OR	95% CI	*P*-value
Male sex	0.73	0.12 - 4.47	0.70
Age	1.26	1.05 - 1.51	0.01
Diabetes mellitus	1.9	0.34 - 10.6	0.50
Atrial fibrillation	1.24	0.20 - 7.58	0.80
History of MI	0.68	0.10 - 4.47	0.70
Stroke	1.32	0.84 - 1.96	0.20
Oncopathology	0.07	0.01 - 2.19	0.13
EuroSCORE II	0.91	0.78 - 1.07	0.30
STS-PROM	1.27	0.74 - 2.19	0.40
LV ejection fraction	0.91	0.84 - 0.98	0.02
Prior MR 2-3	2.45	0.56 - 10.80	0.20
Prior AR 2-3	0.98	0.14 - 7.04	1.00
Prior TR 2-3	0.87	0.03 - 21.6	1.00
Bicuspid AV	3.47	0.26 - 45.70	0.30
Implantation depth	1.24	0.94 - 1.65	0.13
New LBBB	7.09	1.16 - 43.50	0.03
Pre-dilation	0.63	0.13 - 3.09	0.60
Post-dilatation	0.33	0.06 - 1.79	0.20
Procedural failure	5.25	0.33 - 84.1	0.20
Aortic angle	1.00	0.91 - 1.10	1.00

AR=aortic regurgitation; AV=aortic valve; CI=confidence interval; LBBB=left bundle branch block; LV=left ventricular; MI=myocardial infarction; MR=mitral regurgitation; OR=odds ratio; STS-PROM=Society of Thoracic Surgeons Predicted Risk of Mortality; TR=tricuspid regurgitation

## DISCUSSION

Our study identified several key findings: 1) the incidence of new LBBB after TAVR was found to be 20.2%; 2) new-onset LBBB after TAVR was associated with an increased incidence of cardiovascular death; and 3) the development of LBBB was not associated with an increased all-cause death and the need for PPI. LBBB following TAVR is a common complication, likely attributed to the proximity of cardiac conduction system components to the bioprosthesis area (left half of the interventricular septum). Damage to the left bundle branch may result from a combination of patient-related factors (initial conduction disorders, membranous septum length, aortic root calcification severity) and procedural variables (bioprosthesis type, implantation depth, preor post-dilation).

The incidence of LBBB following TAVR varies between studies, with around one-quarter of cases experiencing the first occurrence of LBBB with the use of first-generation valves^[18^,^19]^. Specifically, the development of LBBB was most commonly observed with CoreValve® self-expanding transcatheter valves (Medtronic Inc., United States of America), with rates ranging from 18% to 65%, compared to Edwards SAPIEN™/SAPIEN XT™ balloon-expandable valves (Edwards Lifesciences, United States of America), where rates ranged from 4% to 30%^[20]^. Data on the occurrence of LBBB with the use of new generation valves are limited, with reported incidences ranging from 12% to 22% following implantation of the Edwards SAPIEN™ valve^[21^,^22]^. Similar results have been observed in studies using Portico™ self-expanding bioprosthesis (St. Jude Medical, United States of America) and next-generation Evolut™ R and Evolut™ R PRO valves (Medtronic Inc., United States of America) with a reported incidence of new LBBB ranging between 18 and 28%^[23^,^24]^. Our findings regarding the incidence of LBBB are generally consistent with the literature and emphasize the relevance of this topic. It is noteworthy that the differences observed in certain parameters among the comparison groups in our study (Table 1) support the potential relationship of LBBB with various procedural and anatomical factors, such as the depth of implantation, the type of bioprosthesis, as well as the membranous septum length. It is important to consider the fact that in more than a third of patients, LBBB that developed after TAVR resolves after discharge from the hospital. Nazif et al.^[7]^ reported LBBB resolved in 42.1% of patients within 30 days after TAVR.

Houthuizen et al. were the first to demonstrate the association of LBBB with increased mortality after TAVR in 2012, and subsequent follow-up data supported these results^[23]^. Nazif and Kim et al.^[7^,^24]^ reported that new-onset LBBB after TAVR increased the incidence of adverse clinical events including all-cause mortality and cardiovascular mortality, rehospitalization, and PPI. A comprehensive meta-analysis involving 9,205 TAVR patients further confirmed the adverse impact of new-onset LBBB on long-term TAVR outcomes at one, two, and three years^[25]^. Our results generally demonstrate similar outcomes. In the new LBBB group, there was a higher incidence of death from cardiovascular causes (HR 2.31, 95% CI 1.18 - 4.53, *P* = 0.012). However, there were no significant differences in the incidence of death from all causes and implantation of permanent pacemaker. The discrepancy between the results regarding the association of LBBB with increased mortality and adverse clinical outcomes may be related with different definitions of LBBB, characteristics of different patient populations, and variability in follow-up time.

The underlying pathogenesis of the detrimental effects of the new-onset LBBB on the long-term outcomes after TAVR is associated with a reduction in LV diastole time, along with abnormal movement of the interventricular septum, leading to a decrease in both regional contractility and global LV EF^[26]^. These findings have been supported by various retrospective studies, which have demonstrated a significant decline in LV EF and a decrease in the effectiveness of LV reverse remodeling processes in individuals with new LBBB^[7^,^9^,^24]^. In the presented study, the analysis of echocardiographic data in the long-term period was omitted due to the lack of available data.

Thus, post-TAVR LBBB represents a significant conduction disorder, mainly due to its high incidence and potential adverse impact on clinical outcomes. It is becoming apparent that in addition to the improvement of transcatheter valves and methods of implantation, optimization of the health care system is required in the context of more thorough postoperative monitoring of this group of patients. In our view, procedural approaches to prevent LBBB after TAVR should involve techniques such as higher implantation of a bioprosthesis (cusp overlap), TAVR without preliminary balloon dilatation (direct implantation), implantation of balloon-expandable valves, and conducting invasive electrophysiological studies alongside the TAVR procedure.

### Limitations

The main limitation of the study is the retrospective design with a relatively small sample size, based on a single-center experience. We also didn't consider the prognostic impact of procedural factors on the development of LBBB and didn't pay attention to cases of resolution of LBBB at different time points in the long-term follow-up. Data on drug therapy that could potentially influence the development of LBBB were also not available. The lack of adjustment for multiple comparison in the multivariate regression analysis may also have partially skewed the results.

## CONCLUSION

New-onset persistent LBBB after a TAVR procedure is associated with an increased incidence of cardiovascular mortality in the long term. However, the occurrence of all-cause mortality and the frequency of PPI are not affected by post-TAVR LBBB.
